# Malignant syphilis in a patient with acquired human immunodeficiency virus (HIV) infection^☆^

**DOI:** 10.1016/j.abd.2021.11.003

**Published:** 2022-07-05

**Authors:** Ana Sofia Pereira, Aluixa Lozada, Ana Filipe Monteiro

**Affiliations:** a Dermatovenereology Service, Hospital de Santarém, Santarém, Portugal; b Anatomopathological Service, Hospital de Santarém, Santarém, Portugal; c Dermatovenereology Service, Hospital Garcia de Orta, Almada, Portugal

**Keywords:** HIV, Syphilis, *Treponema pallidum*

## Abstract

Malignant syphilis is an uncommon variant of syphilis, most often (but not always) found in immunosuppressed individuals. This report describes the case of a 57-year-old man, infected with the acquired human immunodeficiency virus (HIV), with a generalized picture of erythematous-squamous papules that rapidly progressed to painful and ulcerated plaques and nodules, some covered with a black rupioid crust. The analytical study performed revealed positive VDRL (Venereal Disease Research Laboratory) and RPR (Rapid Plasma Reagin). The skin biopsy was nonspecific; however, the immunohistochemical analysis disclosed the presence of spirochetes. The patient was then treated with benzathine penicillin G 2.4 MU once a week IM for three weeks, with progressive resolution of the lesions. Considering its rarity, this atypical form of syphilis that needs to be known to better recognize its clinical presentation and provide more prompt treatment to patients.

A 57-year-old human immunodeficiency virus (HIV) positive patient came to the Department of Dermatology due to a symmetrical and generalized dermatosis consisting of erythematous-squamous papules with one-month evolution (Fig. 1A), which gradually progressed to painful ulcerated plaques and nodules, some covered with lamellar and adherent crusts (Fig. 1B‒C). The lesions were found on the scalp, face, trunk and also on the limbs. The palms, soles and mucous membranes were spared. The patient also reported fever, night sweats, and non-quantified weight loss in the previous weeks. He denied having risky sexual behaviors and declared having had a single sexual partner in the last 10 years.

**Figure 1:**
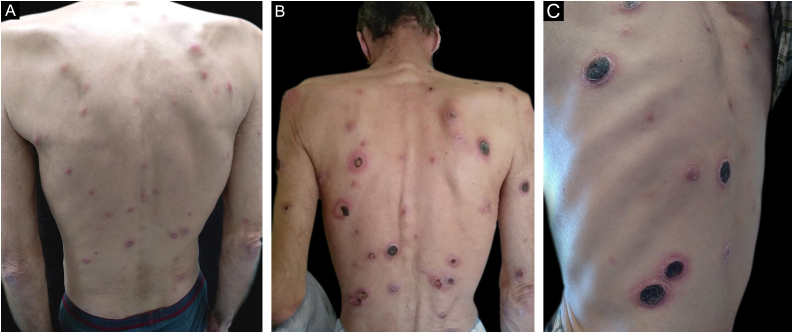
Clinical aspect of the lesions (A) initial erythematous-squamous papules, (B) ulcerated plaques on the trunk (C) lesions covered with rupioid crusts at higher magnification.

The physical examination revealed bilateral inguinal adenomegaly, with no genital or perineal lesions. The neurological examination was normal.

The laboratory tests revealed a viral load of <20 copies/μL and a CD4+ cell count of 507 μL. The VDRL (Venereal Disease Research Laboratory) test was positive at 1:32 dilutions, as was the RPR (Rapid Plasma Reagin), at a titer of 1:128.

A skin biopsy was performed, which revealed a lymphohistiocytic infiltrate in the superficial dermis, and no plasma cells or eosinophils were observed.

Immunohistochemistry for spirochetes showed the presence of numerous ‘corkscrew-shaped’ microorganisms, compatible with *Treponema pallidum* (Fig. 2).

**Figure 2:**
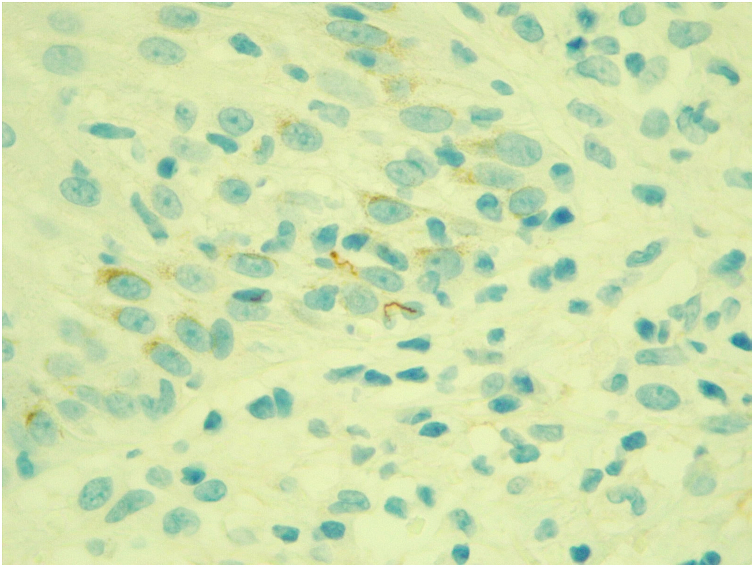
Immunohistochemical examination positive for *Treponema pallidum* (×630).

The diagnosis of malignant syphilis was established and an intramuscular injection of benzathine penicillin G 2.4 MU was administered once a week, for three weeks. There was no Jarisch-Herxheimer (JH) reaction. The skin lesions gradually resolved, with residual hypopigmented scars.

Malignant syphilis (MS), also known as lues maligna or rupioid syphilis, is an uncommon form of secondary syphilis.^1^ Co-infection with HIV seems to be an important predisposing factor for MS, as these patients are 60-fold more likely to have this form of syphilis.^2^

Low CD4 counts may also favor MS, as most patients with HIV and MS have CD4 counts <500 cells/μL.^1^ However, there have been reported cases of MS in immunocompetent individuals,^3^ as well as in HIV-positive individuals with normal CD4 counts,^4^ as the patient described in the present case.

The classic clinical presentation of MS consists of squamous or crusted papules and plaques that become ulcerated or necrotic.^1^

Due to the nonspecificity of the lesions, the differential diagnosis must include several entities, such as pyoderma gangrenosum, vasculitis, lymphoma, pityriasis lichenoides, erythema necrotisans and ecthyma gangrenosum.

The criteria developed in 1969 and used to the present day to aid in the diagnosis of MS include (1) Compatible clinical and microscopic aspects; (2) High serological titer for syphilis; (3) Severe JH reaction; and (4) Excellent response to antibiotic therapy.^5^

In the present case, the diagnosis of MS was confirmed by the suggestive clinical condition, the positive VDRL and RPR tests, and the rapid resolution with the administration of penicillin. Additionally, the identification of *Treponema pallidum* by immunohistochemistry was crucial, given the nonspecificity of the histological findings, namely the absence of plasma cells.

Although uncommon, MS is a clinical entity that should be promptly recognized by dermatologists and general practitioners, as its early diagnosis and treatment result in less morbidity and better control of the dissemination of the infection.

## Financial support

None declared.

## Authors' contributions

Ana Sofia Pereira: Drafting and editing of the manuscript; literature search; approval of the final version of the manuscript.

Aluixa Lozada: Analysis and interpretation of data; approval of the final version of the manuscript.

Ana Filipe Monteiro: Critical review of the content; approval of the final version of the manuscript.

## Conflicts of interest

None declared.
